# A flexible, interactive software tool for fitting the parameters of neuronal models

**DOI:** 10.3389/fninf.2014.00063

**Published:** 2014-07-10

**Authors:** Péter Friedrich, Michael Vella, Attila I. Gulyás, Tamás F. Freund, Szabolcs Káli

**Affiliations:** ^1^Laboratory of Cerebral Cortex Research, Institute of Experimental Medicine, Hungarian Academy of SciencesBudapest, Hungary; ^2^Faculty of Information Technology, Péter Pázmány Catholic UniversityBudapest, Hungary; ^3^Department of Physiology, Development and Neuroscience, University of CambridgeCambridge, UK

**Keywords:** neuronal modeling, python, software, simulation, model fitting, parameter optimization, graphical user interface

## Abstract

The construction of biologically relevant neuronal models as well as model-based analysis of experimental data often requires the simultaneous fitting of multiple model parameters, so that the behavior of the model in a certain paradigm matches (as closely as possible) the corresponding output of a real neuron according to some predefined criterion. Although the task of model optimization is often computationally hard, and the quality of the results depends heavily on technical issues such as the appropriate choice (and implementation) of cost functions and optimization algorithms, no existing program provides access to the best available methods while also guiding the user through the process effectively. Our software, called Optimizer, implements a modular and extensible framework for the optimization of neuronal models, and also features a graphical interface which makes it easy for even non-expert users to handle many commonly occurring scenarios. Meanwhile, educated users can extend the capabilities of the program and customize it according to their needs with relatively little effort. Optimizer has been developed in Python, takes advantage of open-source Python modules for nonlinear optimization, and interfaces directly with the NEURON simulator to run the models. Other simulators are supported through an external interface. We have tested the program on several different types of problems of varying complexity, using different model classes. As targets, we used simulated traces from the same or a more complex model class, as well as experimental data. We successfully used Optimizer to determine passive parameters and conductance densities in compartmental models, and to fit simple (adaptive exponential integrate-and-fire) neuronal models to complex biological data. Our detailed comparisons show that Optimizer can handle a wider range of problems, and delivers equally good or better performance than any other existing neuronal model fitting tool.

## Introduction

Currently available experimental data make it possible to create increasingly complex multi-compartmental conductance-based neuron models, which have the potential to imitate the behavior of real neurons with great accuracy (De Schutter and Bower, 1994a,b; Poirazi et al., 2003; Hay et al., 2011). However, these models have many parameters, which are often poorly (or, at best, indirectly) constrained by the available data. One alternative to using detailed biophysical models, which is often used in network simulations, is to utilize much simpler (e.g., reduced compartmental or integrate-and-fire type) model neurons. These have fewer parameters; however, the remaining parameters are often not directly related to the underlying biophysics, and need to be set such that the behavior of the model cell best approximates that of the real neuron (Naud et al., 2008; Gerstner and Naud, 2009; Rossant et al., 2011). In most cases, the relationship between the values of the parameters and the output of the model is nonlinear (for an interesting exception, see Huys et al., 2006) and often rather complex. Accordingly, the task of finding the optimal parameter values is highly non-trivial, and has been the subject of extensive research (Vanier and Bower, 1999; Keren et al., 2005; Huys et al., 2006; Druckmann et al., 2007, 2008; Gurkiewicz and Korngreen, 2007; Van Geit et al., 2007, 2008; Huys and Paninski, 2009; Rossant et al., 2010, 2011; Eichner and Borst, 2011; Hendrickson et al., 2011; Bahl et al., 2012; Svensson et al., 2012; Vavoulis et al., 2012).

These studies have proposed a variety of methods to find the best-fitting model; the main differences concern the way in which the output of the model is compared to the target data (the cost function), and the procedure used to come up with new candidate solutions (the optimization algorithm). There are also several existing software solutions to this problem; notably, the general-purpose neural simulators NEURON (Carnevale and Hines, 2006) and GENESIS (Bower and Beeman, 1998) both offer some built-in tools for parameter search (Vanier and Bower, 1999), and some programs [such as Neurofitter (Van Geit et al., 2007) and Neurotune^1^] have been specifically developed for this purpose. However, most of these tools offer a very limited choice of cost functions and/or optimization algorithms (and adding new ones is typically not straightforward), and thus it becomes difficult to apply them to new scenarios and to take advantage of new developments. In addition, few of these existing tools offer an intuitive user interface which would guide the casual user through the steps of model optimization, although an increasing number of laboratories now use computer simulations to complement experimental approaches, and employ model-based techniques to extract relevant variables from their data, which typically require the fitting of multiple model parameters.

In this article, we describe a new software tool called Optimizer^2^, which attempts to address all of these issues. It offers an intuitive graphical user interface (GUI), which handles all of the main tasks involved in model optimization, and gives the user access to a variety of commonly used cost functions and optimization algorithms. At the same time, it is straightforward to extend the capabilities of the program in many different ways due to its modular design, which allows more advanced users to adapt the software to their particular needs.

## Design goals and principles

The full specification of a model optimization problem requires one to provide the following pieces of information: (1) the form of the model, both at an abstract level (e.g., multi-compartmental model with a given morphology and channel specifications, or integrate-and-fire model of a given type) and as a specific implementation (e.g., a set of.hoc and.mod files in NEURON); (2) the set of tunable parameters in the model (along with their possible ranges); (3) the simulation protocol, i.e., the way the model should be stimulated and the variables to be recorded; (4) the target data (from experiments, or sometimes from a different model); (5) the cost function, i.e., a measure of how different the output of a particular simulated model is from the target data (this may be as simple as the sum of squared error over corresponding data points, or may involve the extraction and comparison of various features from the simulations and the target data). If there are multiple error measures (objectives), one possible approach, called single-objective optimization, is to define a single combined cost function by appropriately weighting the different objectives. Another approach, known as multi-objective optimization, is to treat each error measure separately, and look for a whole set of optimal solutions which represent different trade-offs between the objectives. Once the problem has been fully specified, the last critical ingredient is the algorithm which attempts to solve the model optimization problem by finding the set of tunable parameters which minimizes the cost function, i.e., the parameters for which the output of the model is as similar as possible to the target data. For relatively simple problems, several common algorithms will be able to find the single best set of parameters to a high degree of precision in a relatively short time; for very complex problems, no algorithm can be guaranteed to find this global optimum in a reasonable amount of time. In these latter cases, different optimization algorithms use qualitatively different strategies to come up with good solutions (which may or may not be the globally optimal one). Local algorithms (such as gradient descent) find the best solution in a limited part of the parameter space (typically defined by the initial set of parameters from which the search begins); global algorithms (such as evolutionary algorithms and simulated annealing) employ various heuristic strategies to explore the parameter space more extensively, while taking advantage of intermediate results to come up with new promising candidate solutions.

All of the components above may have almost infinitely many variants, so it may seem hopeless to create a simple user interface which allows one to specify such a large variety of problems effectively. However, several facts help alleviate this problem to some extent. First, a large percentage of the use cases that occur in practice are covered by a limited set of components; for instance, many electrophysiological experiments apply either current clamp or voltage clamp configurations with step stimuli while recording the membrane potential or holding current, respectively. These common situations can be managed effectively from a GUI. Second, for the models themselves, which show the largest possible variability, there are widely used structured descriptions, partly in generic formats [such as NeuroML (Gleeson et al., 2010) and NineML^3^], and partly in the form of model definitions in the languages of neural simulators (such as NEURON's.hoc and.mod files). These descriptions may be read and manipulated by the model optimization software, and also directly lead to code which may be executed by the simulators. Finally, the nature of the task is modular; the same ingredients may be combined in many different ways so that it becomes possible, for example, to implement a new cost function and then use it in combination with existing models, simulation protocols, and optimization algorithms.

Following these considerations, we set two (apparently conflicting) goals for our implementation of model optimization. On one hand, we wanted our solution to be as flexible as possible, supporting a wide and easily extensible range of model types, modeling software, simulation protocols, cost functions, and optimization algorithms. On the other hand, we wanted to provide a simple and intuitive interface which would guide the user through a variety of commonly occurring model optimization scenarios, without requiring programming expertise or deep knowledge of the optimization process.

In order to attain both of these goals, we decided to implement an essentially two-level design. The lower level (back end) would define the components of the model optimization workflow as described above, as well as the ways these must interact with each other to solve the task. This would be implemented in a highly modular fashion to allow the independent replacement of individual elements as well as the straightforward addition of new elements. The higher level (front end) would be implemented primarily as a GUI (although a command-line interface would also be provided to allow non-interactive processing). The front ends would allow the user to select from the components provided by the back end, and to set all relevant options and parameters within these components. In addition, the GUI would also provide some basic tools for the inspection of data and the results of model optimization.

## Implementation

The Python programing language was the obvious choice for the implementation of the software. First, Python offers the necessary power and flexibility to handle the task. Second, the open source modules offered by Python already include solutions to many important sub-tasks, such as data handling, visualization, and non-linear optimization. Almost all of the commonly used neural simulation packages now have a Python interface (Eppler et al., 2008; Goodman and Brette, 2009; Hines et al., 2009; Cornelis et al., 2012). This makes Python an optimal tool for the creation of the aforementioned framework.

The software can interface directly with NEURON to read, modify, and run models described in NEURON's own format. Other simulators are supported indirectly as “black boxes” which communicate with Optimizer through files, and return simulation results based on parameters generated by Optimizer. Optimization itself can be carried out using a selection of (local and global) algorithms from the inspyred^4^ and scipy^5^ packages. Most cost functions are implemented within Optimizer, except for Phase Plane Trajectory Density (PPTD), which relies on the pyelectro package^6^. The GUI was implemented using the wxPython package^7^, a wrapper for the C++ GUI toolkit wxWidgets.

**Figure 1:**
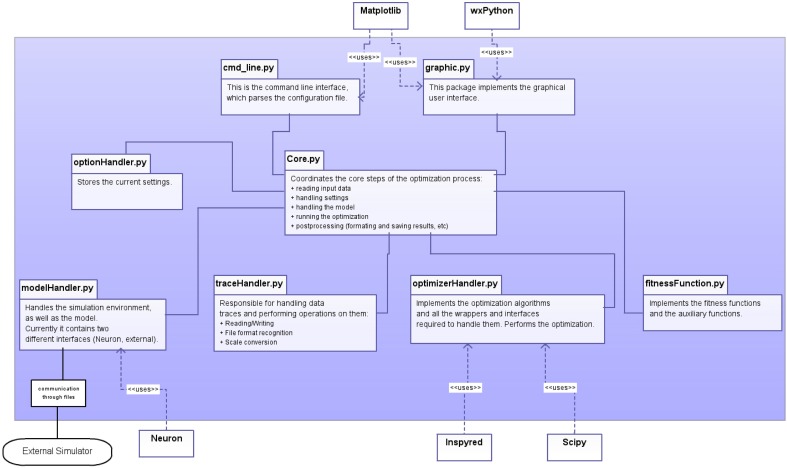
**Schematic representation of the design of the software, showing the main components of Optimizer (within the blue shaded area), interactions among its modules and with critical external modules**.

The program has a modular structure, and each module handles distinct tasks (Figure 1). The modules are the following:

1. Core module:
  This is the main module for the software. It interacts with all the other modules, and performs the necessary steps of model optimization via the methods of the *coreModule*:
  - reading input data
  - loading the model file and selecting the parameters subject to optimization
  - setting up the stimulation and recording protocol
  - selecting a fitness function (or a weighted combination)
  - selecting the algorithm with different parameters and performing the optimization
  - storing the configuration file and saving the results of optimization in various formats
2. traceHandler module:
  Contains the main data holder class, called *Data*, which encapsulates the *Trace* class which is responsible for the handling of an independent trace set. The *Data* class is also responsible for reading the input data files appropriately. The traceHandler module also contains functions performing subtasks related to data handling, such as unit conversion. Currently the *Data* class can handle only one set of data of identical types (e.g., a series of voltage traces of given length), but we are planning to support multiple sets of data with different types (e.g., voltage traces plus explicit spike times) as well as abstract inputs (such as the values of extracted features).
3. modelHandler module:
  Contains two main classes which are responsible for handling neuronal models. The first one, called *modelHandlerNeuron*, is responsible for the built-in support of the NEURON environment, and handles the various stimulation protocols and recording methods which are directly accessible for models implemented in NEURON, as well as parameter assignment and other model-related tasks. The second class, called *externalHandler*, is responsible for the handling of external, user-specified simulators.
4. optionHandler module:
  A simple container class to hold the specified settings. This class can also read and write a configuration file.
5. optimizerHandler module:
  This module contains the implementations of the different optimization algorithms as separate classes, along with assorted auxiliary functions such as parameter normalization, boundary selection, etc.
  The user can extend the list of algorithms by implementing a new class within this module and adding a single line to the Core module. To make the new algorithm available via the GUI one must add the name of the algorithm to the appropriate list.
  Five different algorithms are currently implemented, including a customized evolutionary algorithm and a simple simulated annealing algorithm from the inspyred package, as well as the scipy implementations of simulated annealing, the downhill simplex method, and the L-BFGS-B algorithm (see next section for details).
6. fitnessFunctions module:
  Contains the class responsible for implementing and using the different cost functions (fitness functions). The module also contains a class to handle spike objects, which are used in various cost functions. To extend the list of available functions the user can implement his/her own function here as a class method. To make the new function available, the user must add the alias name-function object pair to the list of cost functions, and add the alias name for the function which will appear in the GUI to the Core module.
  The currently available cost functions are the following (see next section for detailed descriptions): mean squared error, mean squared error excluding spikes, spike count, spike count during stimulus, ISI differences, latency to first spike, AP overshoot, AP width, AHP depth, derivative difference, PPTD. The PPTD method is available through the external pyelectro module, while the rest are implemented by Optimizer.
  As the program supports arbitrary combinations of these cost functions, the main method in this class is the *combineFeatures* function, which creates the appropriate weighted combination of the given fitness functions, and calculates the accumulated fitness value over the corresponding pairs of traces in the simulated and target data sets.

## Program capabilities and basic usage

Depending on the exact needs and degree of expertise of the user, the software can be used at three different levels of complexity. At the simplest level the user can perform optimization tasks using the built-in tools of the graphical interface or run the optimization from the command line using a configuration file. At the next level the user can extend various capabilities of the GUI by using external files (see below). At the most advanced level the user can construct his/her own optimization framework using the building blocks provided by the package, or extend its functionality by adding new algorithms or fitness functions. To support this last level, we concentrated on structural simplicity while creating the modules.

As we briefly mentioned earlier, model implementations for certain simulators (currently NEURON) can be handled, interpreted, and modified internally by Optimizer (“internal simulators”), while models designed for other simulators can be optimized as “black boxes” (i.e., only looking at their inputs and outputs), and only if they provide their own interface to Optimizer (by taking inputs and producing outputs in the format expected by Optimizer; see Appendix). These “external simulators” must take care of setting up the simulations (including the model itself, but also the stimulation and recording protocols), but they can still take advantage of the variety of cost functions and powerful optimization algorithms provided by Optimizer. Internal simulators are supported at a much higher level; in particular, their internal parameters can be viewed and selected for optimization, and several common simulation protocols (such as current and voltage clamp recordings using step stimuli) can be set up directly from the Optimizer GUI.

There are two parts of the specification of the model optimization problem where several commonly occurring scenarios are difficult to capture by a few discrete choices and continuous parameters, and are thus inconvenient to control entirely from a GUI. First, while the GUI allows the user to select for optimization any combination of the parameters of a NEURON model, this does not cover the frequent cases where multiple model parameters (at the level of the actual implementation) are controlled by a single (or a few) abstract parameters, or there are other kinds of joint constraints on the model parameters. For example, when we wish to determine the passive biophysical properties of a realistic multi-compartmental model based on the measured response to injected current, we normally do not want to consider the membrane resistance values of all the dendritic sections as independent parameters (which would lead to a very high number of free parameters and an intractable optimization problem); instead, we take as the free parameter the value of the specific membrane resistance, and calculate the corresponding values of the actual membrane resistance (or leak conductance) in each part of the cell based on the measured geometry. In order to allow the distinction between the (potentially abstract) parameters set by the optimization algorithms and the actual parameters of a particular model implementation, and to allow the implementation of an arbitrary mapping between the two, we introduced “user functions” into our model optimization framework. These user functions define the (abstract) parameters to be optimized, and also define (using NEURON's Python interface) how these abstract parameters should control the internal parameters of the NEURON simulation (see Appendix for details). This solution also makes it possible to optimize parameters of the simulation which are not strictly part of the model neuron (such as the properties of incoming synaptic connections, as demonstrated by one of the use cases described below).

Second, while current and voltage steps are fairly common ways of stimulating a neuron, and their characteristics are easily specified by a handful of parameters which are straightforward to control from a GUI or a configuration file, many other stimulation protocols are also widely used in both experiments and simulations to characterize the behavior of neurons in different ways. Some of these protocols (such as sine wave and noise stimulation) will probably be added to the GUI in the future. Meanwhile, we opted for the more generic approach of allowing input whose time dependence is given explicitly in an external file (see Appendix). We demonstrate the utility of this approach in one of the use cases described below, where input to the neuron consisted of two consecutive current pulses of different duration and amplitude.

One of the most critical choices in setting up a model optimization problem involves the cost function (or fitness function), as this choice (along with the simulation protocol) determines the sense in which the behavior of the optimized model neuron should be close to that of the target neuron. The importance of this choice is also reflected in the large variety of different cost functions which have been proposed, and we aimed to provide access to many of these within Optimizer. The software currently supports the following cost functions (full details can be found in the package reference part of the online documentation):

Mean squared error: the mean squared difference of the two traces in a point by point manner, normalized by the squared range of the experimental data.
Mean squared error excluding spikes: the same as above, but compares only the subthreshold part of both traces, excluding parts of both traces in time windows of a given width around each spike.
Derivative difference: the normalized average squared difference of the temporal derivatives of the given traces.
Spike count: the absolute difference in the number of spikes in the entire trace, normalized by the sum of the two spike counts (plus one, to properly handle the case with no spikes)
Spike count during stimulus: the same as above, but only takes into account spikes which occur during the time of the stimulus.
ISI differences: the sum of the absolute differences between the corresponding inter-spike intervals in the two traces, normalized by the length of the traces.
Latency to 1st spike: the squared difference in the latency of the first spikes, normalized by the squared length of the traces.
AP overshoot: the average squared difference of action potential amplitudes, normalized by the squared maximal AP amplitude of the experimental trace. AP amplitude is defined as the difference of the AP peak voltage and the AP threshold.
AP width: the average squared difference of the width of APs, normalized by the squared average width of experimental APs.
AHP depth: the squared average of the differences in after-hyperpolarization depth, normalized by the squared range of subthreshold potential in the target trace.
PPTD: Compares the two traces in the phase plane using the method proposed by Van Geit et al. (2007), as implemented by the *pptd_error* function from the pyelectro package.

Many of these cost functions have associated parameters which may be set by the user (although sensible default values are also provided). For instance, several cost functions require the detection of spikes, and these allow the setting of the action potential detection threshold, while the subthreshold version of the mean squared error cost function also allows setting of the width of the exclusion window around each spike.

Optimizer also supports arbitrary linear combinations of these cost functions. In order to ensure that the weights given actually correspond to the relative importance of the component cost functions in determining the final cost value, all individual cost functions are normalized in appropriate ways such that their possible values are (at least approximately) in the 0–1 range, as described above. When the input data and the corresponding simulation results consist of multiple traces, the cost functions return the sum of the cost values over the corresponding pairs of traces.

As the implemented functions all use pointwise comparisons at some stage of the calculations, we had to guarantee that the appropriate points are compared. This becomes a problem when the user wants to compare two traces sampled at different frequencies (these traces would have different numbers of points but correspond to the same length of time). We solved this issue by applying the following rules:

If the sampling frequency of the input is higher than the model's sampling frequency, then the simulation time step is adjusted appropriately. If the sampling frequency of the input is lower than the model's sampling rate, then the input is re-sampled at the model's sampling frequency using linear interpolation. Note that, after re-sampling, the program considers the re-sampled trace to be the input trace, and if the original data are required for any reason, they must be reloaded.

Although a very large selection of algorithms have been proposed for the solution of nonlinear optimization problems, we decided to focus (at least initially) on methods which have proved to be efficient for neuronal model fitting. In particular, both evolutionary (genetic) algorithms and simulated annealing methods have been used successfully to fit models with up to tens of parameters (Vanier and Bower, 1999), so we included both of them in the list of supported optimization algorithms. In fact, as different implementations can heavily affect performance, we included two different implementations of the simulated annealing algorithm (one from the inspyred and another from the scipy package). In addition to these global optimization methods, we also included two options for local optimization: the classic downhill simplex method, and the L-BFGS-B algorithm, which is considered to be one of the state-of-the-art local methods (Byrd et al., 1995). We found that all the problems we have considered could be solved efficiently using one or more of these methods; however, the program can also be easily extended with additional algorithms. As several algorithms work best when all the parameters to be optimized are of similar magnitude, while the actual parameters may have very different magnitudes, we run the algorithms with normalized parameters (0–1) and pass the re-normalized values to the simulator. By default, the algorithms start from a random point or set of points (within the specified boundaries), but the user can select a specific starting point, which will be the initial point of the algorithm or will be part of the initial set of points. The optimization algorithms currently supported by Optimizer are the following:

### Global algorithms

#### Evolutionary algorithm

Minimizes the error function using a customized evolutionary algorithm, which uses generational replacement with weak elitism (so that the best solution is always retained) and Gaussian mutation in combination with blend crossover (see the documentation of the inspyred package for details). The size of the population (which may be set by the user, and defaults to 100) is constant throughout the process. The mutation rate can also be specified, with a default value of 0.25.

#### Simulated annealing 1

Uses the framework of evolutionary computation (as implemented by the inspyred package with simulated annealing replacement). The parameters which can be adjusted by the user include the number of generations, the rate and standard deviation of Gaussian mutation, the initial temperature and the cooling rate.

#### Simulated annealing 2

Uses a canonical simulated annealing algorithm (Kirkpatrick et al., 1983) (as implemented in scipy). Adjustable parameters include the number of generations, the cooling schedule, the initial and final temperature, the dwell time, the mutation rate, and the error tolerance.

### Local algorithms

#### Downhill simplex method

Uses the Nelder-Mead simplex algorithm (Nelder and Mead, 1965) to find a local minimum of the cost function. The adjustable parameters are the maximum number of iterations, and independent tolerance limits for the input vector and the value of the cost function.

#### L-BFGS-B

Uses the limited-memory Broyden-Fletcher-Goldfarb-Shanno algorithm with bound constraints (L-BFGS-B) (Byrd et al., 1995) to minimize the cost function. The maximum number of iterations and the target accuracy can be set by the user.

### Usage of the graphical interface

As briefly discussed above, all the basic functionality of Optimizer, along with many of its more advanced features, can be accessed conveniently from a GUI. The GUI consists of seven so-called layers, which are responsible for guiding the user through the steps of the optimization process. A detailed guide to the GUI, with screenshots and explanations of all of its components, is available online from the documentation page of Optimizer^8^, and is also included with the software itself, so only a brief summary will be provided here. The graphical interface can be started from the command prompt with the line:

python optimizer.py-g

Once the program has started, the first layer will appear, where the user can select the file containing the input trace(s). The user must specify the path to this file, and the working directory (base directory) where the output of the program will be written. In addition, the user must provide the type and basic characteristics of the trace set. After loading the selected file, the traces are listed in a tree display, and their successful loading can be verified in a plot which displays all the traces concatenated (concatenation is performed only for displaying purposes, and the traces are otherwise handled separately).

On the second layer the user can specify the simulator (currently NEURON or “external,” see above). With NEURON as the simulator, the model can be loaded simply after selecting the main.hoc file as the model file; if the model requires.mod files which reside in a different directory, the location of this folder must also be provided. The model file should contain only the specification of the neuron and the necessary mechanisms. We note that, in the current version of Optimizer, loading a model (.hoc file) whose special mechanisms (compiled.mod files) cannot be found, leads to a situation from which the program cannot recover the correct model, and the software should be restarted.

**Figure 2:**
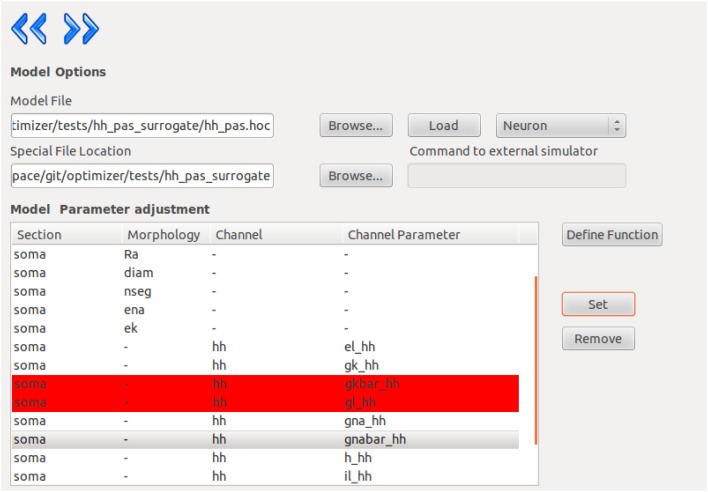
**Screenshot from the Optimizer GUI, showing the model selection and parameter selection interface**.

Once the model is loaded successfully, the content of the model will be displayed, and the user can select parameters by picking them in the list and pressing the “set” button. Removing a parameter is done in a similar fashion (Figure 2).

As mentioned earlier, the functionality of the GUI can be extended by using external files. The second layer also allows the user to load or define the “user function” which defines a set of abstract parameters to be optimized, and describes the mapping from these abstract parameters to actual parameters of the model implementation (in NEURON).

The next layer specifies the simulation protocol, and contains the settings regarding stimulation and recording. The user can select the stimulation protocol which can be either current clamp or voltage clamp. The stimulus type can also be selected (currently, either step protocol or custom waveform). If the step protocol is selected, the properties of the step can be specified. Multiple stimuli of different amplitudes can also be specified; via the GUI, the user can provide up to 10 stimulus amplitudes. If custom waveform is selected as stimulus type, the time course of the stimulus can be loaded from an external file specified by the user. Finally, the user must choose a section and a position inside that section to stimulate the model.

In the second column of this layer, the parameters controlling the simulation and the recording process can be given. The user must give an initial voltage parameter, the length of the simulation and the integration time step (variable time step methods are not supported yet). The user can select the parameter to be measured (either current or voltage), the section and the position where the measurement takes place.

The next layer is responsible for the selection of the cost function, or combination of cost functions with the desired weights. Optimizer offers weight normalization with the press of a button, but unnormalized values are accepted as well. The user can fine-tune the behavior of the cost functions by giving parameters to them (the value of the same parameter should be the same across the functions).

On the next layer, the user can select the desired optimization algorithm from a list and tune its parameters. The program requires boundaries for all the parameters. The user can also provide initial values for the parameters, which will be interpreted differently depending on the algorithm used. In the case of local algorithms, the algorithm will start form the point specified. In the case of global algorithms, the set of values given will be included in the initial set of parameters. At this point, the model optimization problem is fully specified, and optimization will start when the Run button is pressed.

After the program finished the optimization process, the result can be viewed and compared to the target data in a graph. This result is then saved into a text file, picture files in.png and.eps formats, and into an HTML file. The text file contains the data trace(s) obtained from the model by running the simulation with the optimal parameters. The picture shows the target and the resulting trace for visual comparison. The HTML file serves as a report, as it contains the most important settings of the optimization process as well as the resulting parameter values and a plot of the target and result trace. The program also saves all the settings required to reproduce the optimization process in a configuration file in XML format, which can also be used to run the optimization using the command-line interface (see below).

The last layer offers some additional tools to analyze the results. Here, the software displays the basic statistics of the last population of results. If one of the algorithms from the inspyred package (such as the evolutionary algorithm or its implementation of simulated annealing) was used, a *generation plot* (which displays the change in the cost value from generation to generation) and an *allele plot* are also available.

**Figure 3:**
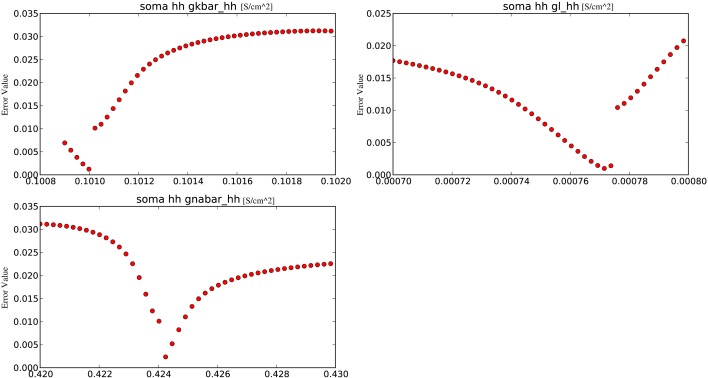
**An example of the grid plot of Optimizer, showing one-dimensional slices of the error as a function of the parameters, in the vicinity of an optimum found by the software**.

The final analytical tool offered by Optimizer is the *grid plot* which evaluates and plots the cost function over a set of parameters, thus allowing the user to observe a part of the search space (Figure 3). The parameter set is created by fixing every parameter except one to their optimal values and allowing the remaining one parameter to vary. By repeating this process for every parameter, we obtain one-dimensional slices of the cost function around the optimum. Ranges for the grid plot are initialized to the boundaries of the search space defined earlier, but they can be reset to wider or narrower ranges (the latter can be useful to observe the close proximity of the optimum), providing an insight into the model's parameter sensitivity.

### Usage of the command line interface

The command line interface can be started similarly to the GUI (with a different option), but requires an additional argument, the name of the configuration file. The program can be started by typing:

python optimizer.py-c conf.xml

The configuration file must be an XML file, which contains the settings for the optimization process in XML tags. Every option has its own XML tag and the value between the tags must be in the appropriate format for the software to recognize. This feature was added to support systems where a graphical interface is not needed or not available (the optimization must run without user interaction).

As this interface is considered an auxiliary one, it currently has no error detection implemented; e.g., a missing parameter will be detected only during runtime. Thus we recommend that the user generate the configuration file via the GUI by running a simple optimization, and modify the resulting file where necessary.

## Use cases

We designed Optimizer to be able to handle a wide range of model optimization tasks, and we have tested it on a large number of different problems. Model optimization problems can differ in many characteristics (including model type, tunable parameters, simulation protocol, target data, and cost function), and can be attacked using various optimization methods, as described earlier. One important aspect of the problem that we have not discussed is the source and nature of the target data. A traditional way of testing a model optimization algorithm is to generate target data from a model, and then consider some of the parameters to be unknown and attempt to reconstruct their correct values by optimizing the same type of model. This type of target data will be referred to as surrogate data, and tests using surrogate data are useful to debug software, and also to analyze the difficulty of optimization tasks and the power of optimization algorithms. However, it has been pointed out that tests using surrogate data are very artificial in that an exact solution (a parameter combination with zero error) is known to exist, and methods that perform well on surrogate data do not necessarily do well on real data (Druckmann et al., 2008). Therefore, we have tested Optimizer using surrogate data, but also on problems where an exact solution is unlikely to exist. This includes the case where the target data were generated by a more complex model than the one being optimized, and fitting is thus performed as a crucial part of model simplification, and also the case where the target data were recorded in a physiological experiment. Here we present our results on a selection of five problems, chosen primarily to showcase the diversity of tasks that Optimizer is able to solve, but also to highlight the features of the software that enable us to efficiently define and solve these problems. All of the examples were run on standard desktop and laptop PCs running various versions of Linux (for details, see the Installation section of the online documentation). A full optimization run required from a few minutes up to about 2 days, depending on the complexity of the model, the number of iterations, and (in the case of the evolutionary algorithm) the size of the population. All the files required for an exact reproduction of these examples, as well as the results of the optimization runs, have been deposited into the public repository of the software. The simplest way to re-run one of the examples involves using the XML file provided with the command line interface described above.

1. Recovering the correct conductance densities (Na, K, leak) of a single-compartment Hodgkin-Huxley model based on a single (suprathreshold) step current injection.

**Figure 4:**
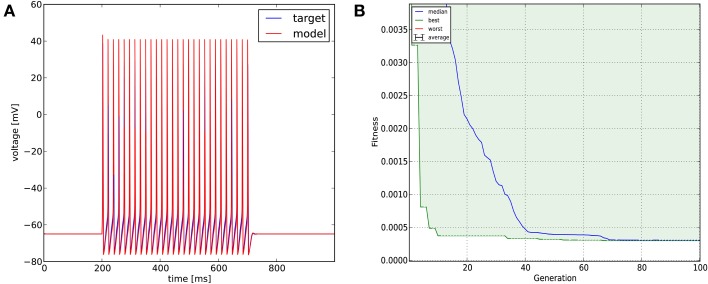
**The results of Optimizer on Use case 1 (fitting conductance densities in the Hodgkin-Huxley model). (A)** Comparison of the original (surrogate) data (blue) and the best-fitting trace (red). Further details of the spike shape are shown in **Figure 9**. **(B)** Evolution of the lowest and median error value across generations in the evolutionary algorithm.

We created in NEURON a single-compartment model containing the original Na^+^, K^+^, and leak conductances of the classical Hodgkin-Huxley model, and set the diameter of the section to 10 μm (the length remained 100 μm, and all the passive parameters and the conductance densities were unchanged). We injected a step current (amplitude = 200 pA, delay = 200 ms, duration = 500 ms) into the soma of this model to create surrogate data (a single 1000 ms long voltage trace). We then changed the densities of the three conductances to create variants of the original model, and tried to find the parameters used for generating the target trace with Optimizer's genetic algorithm (Classical EO; 100 generations with 100 candidates per generation), using the combination of the mean squared error (excluding spikes) and the spike count error functions with equal (0.5) weights. The original and recovered parameters are displayed in **Table 2**. The two traces can be visually compared in Figure 4, which also shows how the lowest value of the cost function changed across generations. It is interesting to note that while the best-fitting trace found matches the target data very well, the algorithm did not manage to recover the exact values of the original parameters. This likely reflects the fact that several different combinations of parameters result in similar voltage traces in response to this current input. This possibility was further investigated by repeating the optimization process using different random seeds, which resulted in different final parameters, but similarly good fits to the data. These findings supported the conclusion that the conductance density parameters of the Hodgkin-Huxley model are not uniquely identifiable using this current injection protocol, but also confirmed that Optimizer was consistently able to find parameter combinations which provide good solutions to this optimization problem.

2. Recovering some basic synaptic parameters from simulated voltage clamp recordings during synaptic stimulation in a single-compartment model.

The target data consisted of the recorded clamp current from a virtual voltage clamp electrode inserted into a single-compartment model, which was essentially the same as the one in Use case 1, and contained Hodgkin-Huxley-type Na^+^, K^+^, and leak conductances plus a conductance-based synapse with a double exponential time course (rise time = 0.3 ms, decay time = 3 ms, maximal conductance = 10 nS, delay = 2 ms). The model neuron received through the synapse a spike train input, which consisted of 4 spikes at regular 100 ms intervals. The task was to recover the four parameters of the synaptic connection.

As we needed to set the parameters of the synapse and the connection (NEURON's NetCon object), and the heuristics used by Optimizer to discover tunable parameters in NEURON models automatically do not cover synaptic parameters (which belong to objects other than the model neuron), we used a simple user function to adjust the parameters. We used the built-in functions of the Optimizer GUI to set up voltage clamp at a constant level (−70 mV); one way to accomplish this is to use a step protocol in voltage clamp with a single amplitude of −70 mV (and arbitrary delay and duration), and an initial voltage of −70 mV.

**Figure 5:**
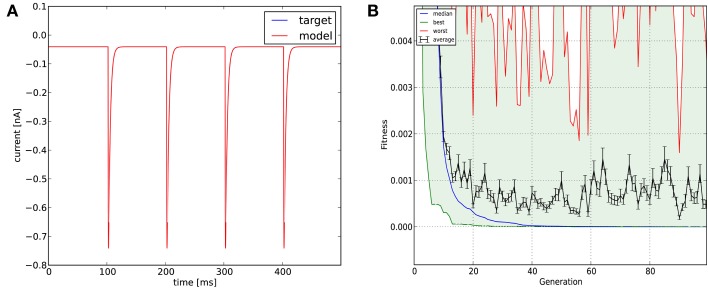
**The results of Optimizer on Use case 2 (fitting synaptic parameters in voltage clamp)**. Figure layout and notation are similar to Figure 4; the error plot in **(B)** also shows the average and worst fitness values for each generation.

Optimization was carried out using the mean squared error cost function. Evolutionary optimization (Classical EO) for 100 generations with a population of 100 restored the original parameters with high precision (**Table 3**; Figure 5). In this case, starting the program with different random seeds always resulted in essentially the same final parameters and consistently low error values.

3. Fitting the densities of somatic voltage-gated channels in a simplified (6-compartment) model to approximate the somatic voltage response of a morphologically and biophysically detailed (CA1 PC) model to a somatic current step, using a combination of features.

The target data trace was obtained from the biophysically accurate and morphologically detailed model of a hippocampal CA1 pyramidal cell (Káli and Freund, 2005) by stimulating the somatic section with a 200 pA step current stimulus. The experiment lasted for 1000 ms and the stimulus started at 200 ms and lasted for 600 ms.

The structure of the simplified model was created before the optimization step by clustering the branches of the detailed model based on the amplitude of the response to subthreshold current stimuli, and combining the branches within each cluster into one compartment of the reduced model. The resulting model had six compartments (one somatic, one basal dendritic, and four corresponding to different parts of the apical dendritic tree). Initial values of the densities of voltage-gated channels in the simplified model were obtained by averaging the corresponding values in the detailed model. The somatic values of the nine channel density parameters were then the subjects of optimization, while dendritic conductance densities, passive membrane characteristics, and geometric properties remained fixed.

**Figure 6:**
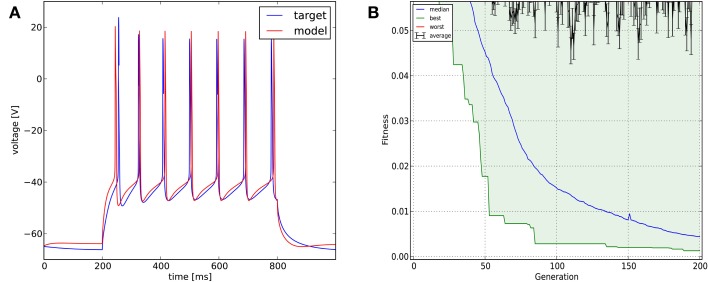
**The results of Optimizer on Use case 3 (fitting voltage traces from a detailed compartmental model)**. Figure layout and notation are similar to Figure 4.

In this case we used a combination of six different cost functions: mean squared error excluding spikes (with weight 0.2), spike count (with weight 0.4), latency to first spike, AP amplitude, AP width, and AHP depth (all four with weight 0.1). The optimization algorithm was Classical EO, and in this case we used 200 generations and 300 candidates per generation to allow a better exploration of the relatively high-dimensional parameter space. The algorithm managed to find a reasonably good solution to this difficult problem, closely matching all of the optimized features (Figure 6; additional details are shown in **Figure 11**).

4. Fitting the passive parameters of a morphologically detailed CA1 pyramidal cell model to experimental data based on a complex current clamp stimulus.

In this case we tried to fit the passive parameters of a morphologically detailed passive model of a hippocampal CA1 pyramidal cell to physiological data recorded from the same neuron (both morphological and physiological data were kindly provided by Miklós Szoboszlay and Zoltán Nusser). The cell was excited by a short (3 ms, 500 pA) and then by a long (600 ms, 10 pA) current pulse (separated by 300 ms) injected into the soma, which is more complex than the simple step stimuli which can be defined using the Optimizer GUI, so we had to use an external stimulus file.

The parameters we were interested in were the specific capacitance and resistance of the membrane and the specific axial resistance. Because we wanted to optimize the parameters cm, Ra, and g_pas in every section of the NEURON model (and also set the e_pas parameter to 0 everywhere in this example, as the baseline voltage had been subtracted from the data), we created a user function to set all the relevant local parameters of the model based on the three global parameters which were optimized.

**Figure 7:**
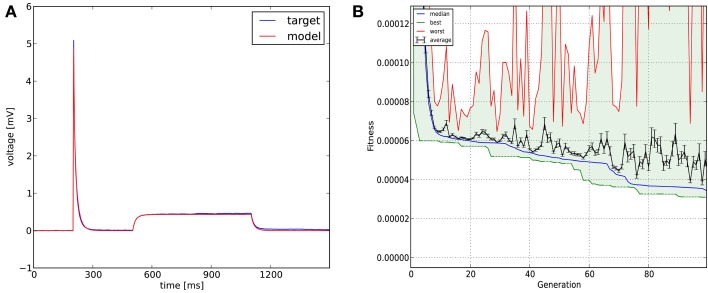
**The results of Optimizer on Use case 4 (fitting the parameters of a morphologically detailed passive multi-compartmental model to experimental data)**. Figure layout and notation are similar to Figures 4, 5. Magnified plots of critical parts of the traces are included in **Figure 12**.

This example demonstrates the importance of the extensibility of the GUI using external files. We used mean squared error as the cost function, and Classical EO; 100 generations and 100 candidates per generation were sufficient to get a good fit to the data (Figure 7; further details of the fit are shown in **Figure 12**).

5. Optimizing the parameters of an abstract (AdExpIF) model to fit the somatic voltage responses (including spikes) of a real cell (CA3 PC) to multiple current step stimuli.

**Figure 8:**
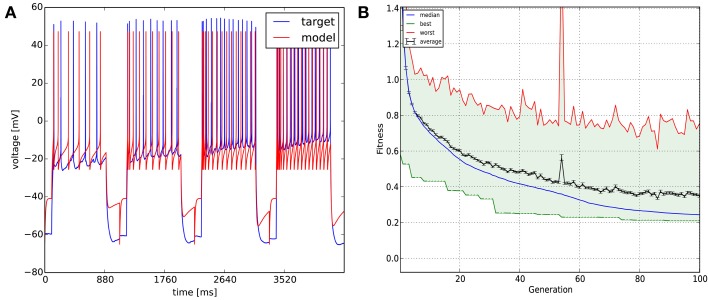
**The results of Optimizer on Use case 5 (fitting an adaptive exponential integrate-and-fire model to experimental data with multiple traces)**. The four traces are displayed in concatenated form in the figure. Figure layout and notation are similar to Figures 4, 5.

In this case we wanted to fit an adaptive exponential integrate-and-fire model to four voltage traces obtained from a real CA3 pyramidal cell. The recordings were 1100 ms long each and the sampling frequency was 5 kHz. The stimulating current amplitudes were 0.30, 0.35, 0.40, and 0.45 nA, respectively. We then optimized the parameters of the model (capacitance, leak conductance, leak reversal potential, threshold voltage, reset voltage, refractory period, steepness of exponential part of the current-voltage relation, subthreshold adaptation conductance, spike adaptation current, adaptation time constant—altogether 10 parameters). As the exponential integrate-and-fire model can be numerically unstable for some combinations of parameters, we had to apply some constraints to the parameters (for example: the spike detection threshold was equal to the spike threshold for exponential calculations plus five times the steepness of exponential approach to threshold). To do this, we created a user-defined function which was loaded by the GUI. We used the combination of the spike count, mean squared error (excluding spikes), latency to first spike, and ISI difference features (which are all meaningful in the context of integrate-and-fire models) with equal weights as the error function, and obtained our results once again using the Classical EO algorithm with 100 generations and 500 candidates per generation (Figure 8). While the resulting model captures the spiking of the neuron relatively well, it clearly cannot deal with the complexities of the subthreshold voltage trace (which is likely due mainly to limitations of the model class itself rather than the fitting process).

## Comparisons with other model optimization tools

### Feature comparisons

Existing publicly available tools for the optimization of neuronal models include NEURON, GENESIS, and Neurofitter. We will now briefly discuss the merits and deficiencies of each of these solutions in comparison to our software.

NEURON features the only GUI-based solution^9^ besides ours, and integrates fully with the most commonly used simulator today. It also includes many useful features, such as the ability to combine results from an arbitrary set of simulations, and to define several regions of interest under visual guidance, which are not yet available in Optimizer. As a consequence, it has been used by several groups, mostly for fitting a few parameters in relatively simple cases. As the Multiple Run Fitter contains only a single relatively basic local optimization algorithm (the principal axis method; Brent, 2002), it may not be suitable for more complex problems. Although an extension to NEURON using genetic algorithms has been developed^10^, it has not been very widely adapted, possibly because (unlike the Multiple Run Fitter and Optimizer) using this extension requires a substantial amount of coding.

The optimization tools of the GENESIS simulator (Vanier and Bower, 1999) cannot be accessed through a graphical interface, and model fitting involves extensive programing in its own script language (whereas, given an implementation of the model itself, optimizing model parameters in Optimizer or NEURON's Multiple Run Fitter requires little or no programming). Thus, implementing a new optimization problem in GENESIS can be quite time-consuming and error-prone. On the other hand, GENESIS implements several powerful optimization algorithms (including customizable versions of a genetic algorithm and simulated annealing) which can produce remarkably good results even by today's standards (the GENESIS implementations are relatively old). Some forms of parallelization are also possible through the PGENESIS module. GENESIS contains a single built-in cost function (a relatively sophisticated algorithm for matching spike times); other error functions need to be added by hand.

Neurofitter is a general-purpose model optimization tool which is in some ways similar to ours (Van Geit et al., 2007). However, Neurofitter does not have a GUI, and the definition of problems needs to be done through a configuration file. It also implements a variety of optimization algorithms, but only a single cost function (the PPTD method), which can be powerful in certain problems, but may be totally inappropriate in other situations. Neurofitter also supports various forms of parallelization through the MPI protocol.

Finally, we note that there are some potentially desirable features which are not currently available in any of the above software solutions (including ours). For instance, multi-objective (rather than single-objective) optimization was found to be advantageous in the context of fitting a full range of models to a diverse set of experimental data (Druckmann et al., 2007), but is not supported by either NEURON, GENESIS, or Neurofitter. Optimizer is also restricted to single-objective optimization for the moment; however, as the inspyred package, one of the main optimization tools used by Optimizer, also supports multi-objective optimization, extending Optimizer to handle this class of problems will be relatively straightforward.

### Performance comparisons

We also wanted to compare, as much as possible, the quantitative performance of different model optimization tools. We therefore attempted to implement the use cases presented earlier for NEURON, GENESIS, and Neurofitter. We did not manage to implement all of the use cases on any of the three other software tools (other than Optimizer). In the end, we successfully completed model optimization in use cases 1–4 using NEURON. We also managed to implement use cases 1–4 using GENESIS, although each of these required a substantial programing and debugging effort; we used the simulated annealing algorithm in this case, as the GENESIS implementation of the genetic algorithm resulted in program crashes on the computers that we used, even for the examples that came with the software. Finally, as we are not aware of any implementation of the AdExpIF model for GENESIS, we could not run use case 5. With Neurofitter, we could run use cases 1–4, while it could not handle the intrinsic numerical instability of the AdExpIF model, and could not complete this optimization without crashing. On the tasks which were successfully solved by several tools, the resulting traces were compared through the mean squared error, and also based on spike count in spiking models (Table 1).

**Table 1:** **Comparison of the error in the best-fitting solution of different optimization software tools on the five problems defined in the Use cases section**.

		**Optimizer**	**Neurofitter**	**NEURON**	**GENESIS**
1—HH	MSE (mV^2^)	0.0033	0.0438	7.32 × 10^−4^	0.0016
	Spike count (28)	28	32	28	28
2—VC	MSE (nA^2^)	1.73 × 10^−7^	0.0052	2.68 × 10^−5^	6.29 × 10^−7^
3—CA1 PC simple	MSE (mV^2^)	0.0069	0.0125	0.0092	0.0028
	Spike count (7)	7	10	6	7
4—CA1 PC morphology	MSE (mV^2^)	3.10 × 10^−5^	3.09 × 10^−4^	3.29 × 10^−5^	3.58 × 10^−5^

*Mean squared error (MSE) was measured in all cases. In problems involving spikes, the resulting spike counts are also shown (the original spike count is shown in parentheses in the second column)*.

**Figure 9:**
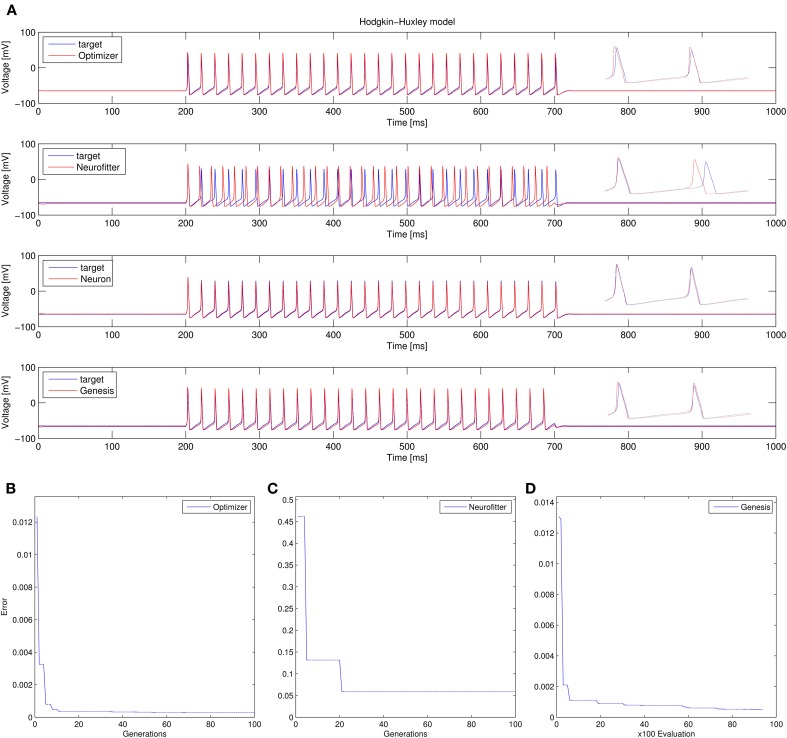
**Comparison of the performance of the four model fitting tools on Use case 1 (fitting conductance densities in the Hodgkin-Huxley model). (A)** Comparison of the resulting best traces with the target trace. Insets show magnifications of spike shapes. **(B–D)** Changes in the lowest error value achieved in each generation (for Optimizer and Neurofitter) or after each 100 model evaluations (GENESIS). Errors are displayed here in arbitrary units, which are different across optimization tools, reflecting differences in the choice of cost functions.

**Figure 10:**
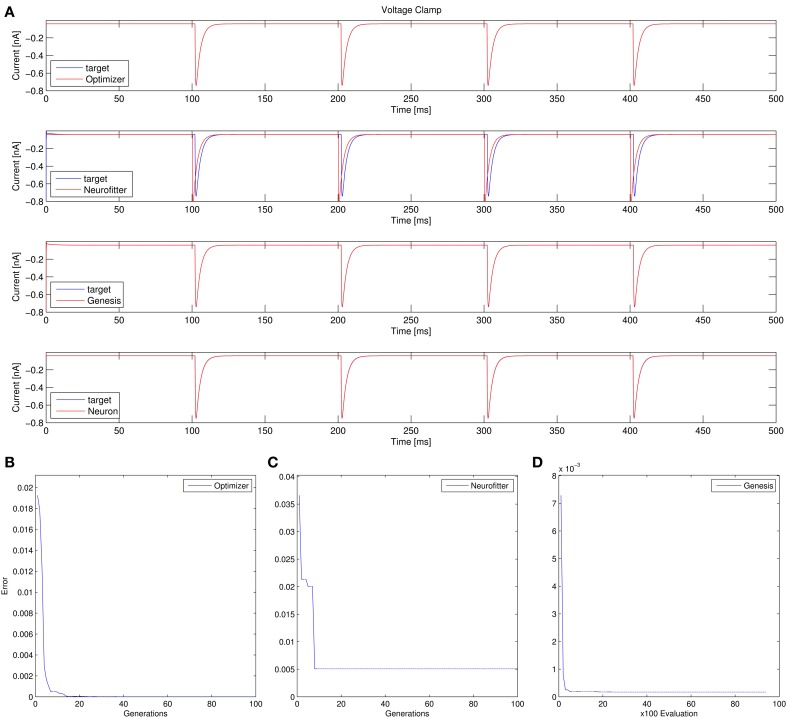
**Comparison of the performance of the four model fitting tools on Use case 2 (fitting synaptic parameters in voltage clamp)**. Figure layout and notation are similar to Figure 9. Error values for Optimizer **(B)** and GENESIS **(D)** reflect mean squared error, measured in (nA)^2^; the value of the PPTD error function in Neurofitter is displayed in **(C)**.

**Figure 11:**
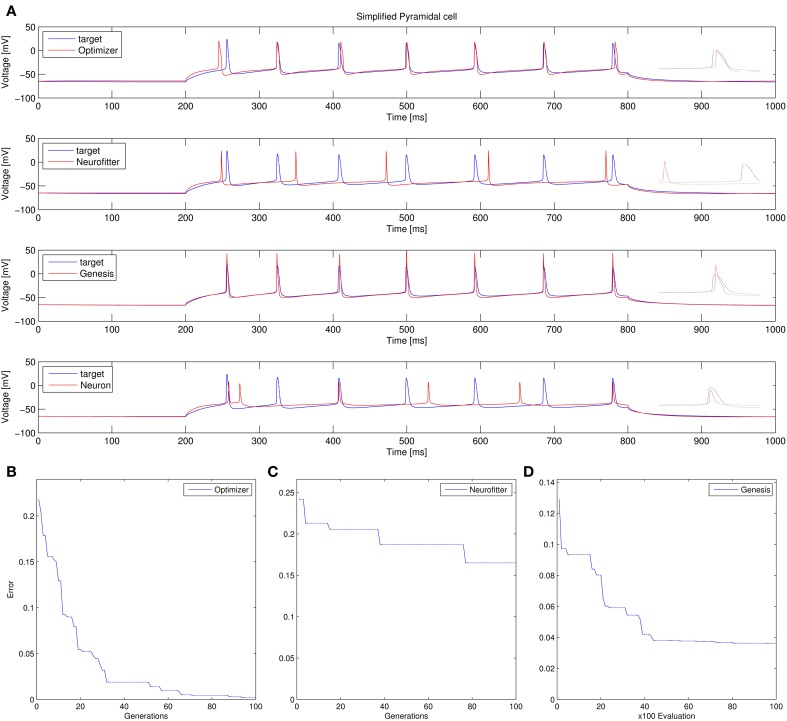
**Comparison of the performance of the four model fitting tools on Use case 3 (fitting voltage traces from a detailed compartmental model)**. Insets allow a better visual comparison of spike shapes. Figure layout and notation are similar to Figure 9. Errors are in arbitrary units, which differ between panels **(B–D)**, due to differences in the cost functions used.

**Figure 12:**
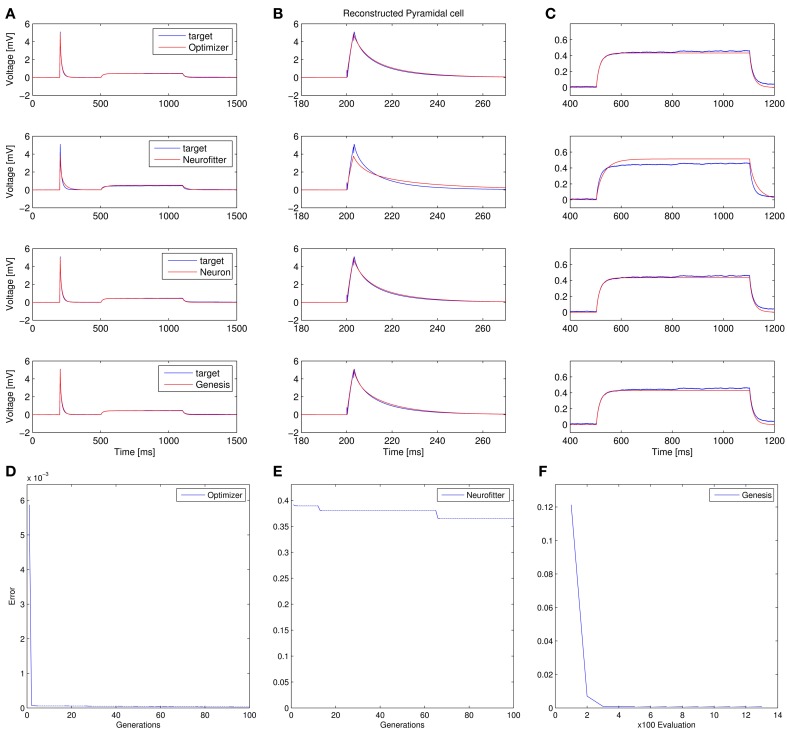
**Comparison of the performance of the four parameter optimization tools on Use case 4 (fitting the parameters of a morphologically detailed passive multi- compartmental model to experimental data). (A–C)** Comparison of the best-fitting traces with the target trace. **(A)** Overview of the whole fit. **(B)** More detailed view of the response to the short input pulse. **(C)** Detailed view of the response to the long pulse. **(D–F)** Plots of the evolution of the lowest error. Error values in panels **(D,F)** reflect mean squared error, measured in (mV)^2^, while PPTD error is shown in panel **(E)**.

1. Recovering the correct conductance densities (Na, K, leak) of a single-compartment Hodgkin-Huxley model based on a single (suprathreshold) step current injection: Three of the four tools (Optimizer, NEURON, and GENESIS) found parameters which resulted in very good fits to the data in terms of spike counts, spike timings, and mean squared error, while Neurofitter found a substantially worse solution (Table 1; Figure 9). However, it is interesting to note that the optimal parameters found by the programs vary significantly among them, and also deviate substantially from the original values (Table 2). This highlights a fundamental issue with the identifiability of the conductance density parameters of the Hodgkin-Huxley model using this current injection protocol.
2. Recovering some basic synaptic parameters from simulated voltage clamp recordings during synaptic stimulation in a single-compartment model: Optimizer and GENESIS could solve this task essentially perfectly, both in terms of mean squared error (Table 1), and in terms of recovering the true values of the parameters (Table 3); Neuron's solution was also close, although slightly less accurate, while Neurofitter's solution had a substantially larger error (Table 1; Figure 10).
3. Fitting the densities of somatic voltage-gated channels in a simplified (6-compartment) model to approximate the somatic voltage response of a morphologically and biophysically detailed (CA1 PC) model to a somatic current step. In order to make the comparison between the different programs as fair as possible, we allowed each program to run for a comparable number of iterations (approximately 10,000); this also meant that, instead of the more extensive optimization allowed for Optimizer whose result was shown in the Use cases section (Figure 6), here we ran Optimizer's Evolutionary Optimization algorithm for only 100 generations with 100 individuals each. On this difficult task, GENESIS came up with the best solution in terms of mean squared error and spike timings (see Table 1), but Optimizer also found a reasonably good solution (with a correct spike count, and a fairly good fit to the subthreshold range, but a worse fit to the actual spike times) (Figure 11). The solutions found by NEURON and Neurofitter were substantially worse, with incorrect spike counts, spike timings, and spike shapes. The different results of the three tools which used global optimization algorithms probably (at least partially) reflect differences in the cost functions used: Neurofitter used its only built-in cost function (PPTD), GENESIS used a combination of mean squared error and its built-in function (spkcmp) for comparing spike timings, while in Optimizer we used a combination of six features (mean squared error, spike count, ISI differences, AP amplitude, latency to first spike, and AHP depth). This example also illustrates that a few properly selected features can result in a solution which is just as good (or better) than one obtained using a larger number of features.
4. Fitting the passive parameters of a morphologically detailed CA1 pyramidal cell model to experimental data based on a complex current clamp stimulus: Optimizer, NEURON, and GENESIS all found approximately equally good solutions (using a mean squared error function), and significantly outperformed Neurofitter (which used PPTD) on this task (Figure 12).

**Table 2:** **Comparison of the best-fitting parameter values with the original values in Use case 1**.

**Parameter**	**Original**	**Optimizer**	**Neurofitter**	**GENESIS**	**NEURON**
gnabar_hh	0.12	0.4242	0.5014	0.2687	0.0968
gkbar_hh	0.036	0.1010	0.1191	0.0714	0.0294
gl_hh	0.000300	0.000769	0.000772	0.000313	0.000320

*Conductance density values are given in S/cm^2^*.

**Table 3:** **Comparison of the best-fitting parameter values with the original values in Use case 2**.

**Parameter**	**Original**	**Optimizer**	**Neurofitter**	**GENESIS**	**NEURON**
tau1 (ms)	0.3	0.3006	0.0406	0.3002	0.2561
tau2 (ms)	3	2.9960	2.9940	2.9998	3.0438
Weight (uS)	0.01	0.010002	0.01209	0.009997	0.010137
Delay (ms)	2	1.9783	0.3672	1.9863	2.0288

In conclusion, Optimizer delivered the lowest or second lowest error (according to our measures) among the four programs tested on all four test cases, and successfully solved a wide variety of problems. While GENESIS could not handle all of these problems (as it does not support integrate-and-fire type models), its simulated annealing algorithm performed very well on the remaining tasks. As we expected, NEURON's local optimization algorithm provided good solutions when the number of parameters was small and the error function contained a single well-defined minimum over a large region of the parameter space, but it performed significantly worse in high-dimensional search spaces, which probably contained multiple local minima. Neurofitter's generally poor performance came as a surprise to us given its demonstrated ability to solve similar problems (Van Geit et al., 2007). However, it is quite possible (even likely) that a better fit could have been achieved with any of these tools by fine-tuning the settings of the optimization algorithms or by using a different cost function, especially on the more complex tasks.

## Future development

This paper describes only a snapshot of the development of our model optimization software. As we demonstrated above, Optimizer is already a working piece of software with many useful functions. Initial development of the program was driven by the realization that no currently available neuronal optimization tool could handle the variety of problems that we encountered in our research, and we are already using Optimizer in the laboratory in several different projects. However, we also aim to provide a tool which is useful for the wider neuroscience community (both the core community of computational neuroscientists and those experimentalists who use modeling as an auxiliary method). Therefore, based on the feedback we receive, we intend to further improve the usability of the program, and also to keep adding features requested by the users. We envisage that some of this development will be handled by the core team of developers (currently three persons), but also hope that the open and modular design of our software will encourage other researchers to contribute and add their favorite protocols, cost functions, and optimization algorithms to Optimizer. We also encourage potential users to send us further use cases, specifying the kinds of model optimization problems that they need solve, so that we can tell them whether and how they can use Optimizer to solve these problems, and to see how we should further extend the capabilities of the program to make it more widely useful. Finally, as Optimizer is released under the GNU Lesser General Public License, it can be used and potentially further developed in other projects.

We have a long and growing list of improvements that we plan to make, and we will describe some of the most important items here. First, as the uniqueness of our software comes mainly from its convenient user interface, we plan to extend the GUI to support an even wider range of problems and options. In particular, as control of the simulations from the GUI is possible only for internal simulators, we aim to support some additional popular simulators (in addition to NEURON) at this level. Adding the simulation platform PyNN (Davison et al., 2008) would be a logical next step, as this would enable us to control all the simulators (including NEST, Brian, and PCSIM) supported by PyNN. Second, we also plan to extend the range of possible target data (and corresponding simulation results) to more complex data sets, possibly including (at the same time) time series (current, voltage, and other continuous variables), discrete events (such as spike times), and abstract (derived) features. This task could be made easier by taking advantage of a Python-based data representation framework such as the neo package (Garcia et al., 2014). Third, we plan to add batch-processing capabilities to the software (first using the command-line interface, but eventually also through the GUI) so that, for instance, the same type of model (with different parameters) could be fitted automatically to data obtained from multiple cells. Finally, as model optimization can be extremely time-consuming, we will look into different ways of parallelizing the process of model fitting. As a first step, we will take advantage of the existing parallel capabilities of the optimization modules (inspyred and scipy) and possibly the simulators themselves. We also plan to make the code within Optimizer more efficient by vectorizing critical calculations (such as the evaluation of the cost functions).

## Conclusions

In this article, we have described a novel tool for the optimization of the parameters of neuronal models. This is a critical, but also complex and often time-consuming step in the construction of biologically relevant models of single neurons and networks. Fitting appropriate models is also becoming an important tool in the quantitative analysis of physiological data sets. However, the results of model fitting can be heavily affected by technical details such as the choice of the optimization algorithm, and actually implementing model fitting has been cumbersome with previously existing tools. This is where we believe our software can make a difference: by making available the power of some of the most advanced methods in model optimization through an intuitive user interface, we hope to make it possible for a larger community of non-expert users to create better models and analyze data in a more efficient and consistent way.

### Conflict of interest statement

The authors declare that the research was conducted in the absence of any commercial or financial relationships that could be construed as a potential conflict of interest.

## Footnotes

^1^http://optimal-neuron.readthedocs.org/en/latest/

^2^Software available at https://github.com/vellamike/optimizer, online documentation at http://optimizer.readthedocs.org/

^3^http://software.incf.org/software/nineml

^4^http://inspyred.github.io/

^5^http://www.scipy.org/

^6^http://pyelectro.readthedocs.org/

^7^http://www.wxpython.org/

^8^http://optimizer.readthedocs.org/en/latest/

^9^http://www.neuron.yale.edu/neuron/static/docs/optimiz/main.html

^10^http://senselab.med.yale.edu/simtooldb/ShowTool.asp?Tool=102464

## Appendix: extending optimizer (technical details)

### Using an external simulator

In the model selection layer, the external simulator option can be selected. In this case a command line statement should be entered into the appropriate box, which consists of the following:

– the command that calls the simulator
– the name of the model file
– options to the simulator (optional)
– as the last parameter, the number of parameters subject to optimization

In order to use an external simulator, the model file must be modified to contain the stimulation and recording protocol and to be able to take input parameters from a text file called “params.param” located in the base directory, which should contain one parameter value on each line. The model must also be able to write the traces resulting from running the simulation to a file called “trace.dat” located in the base directory, in a text file containing TAB-separated numerical values, with each trace in a separate column.

### User function

Also in the model selection layer, the user can define a function which will be executed for every set of parameters, and maps the parameters optimized to the appropriate parameters of the NEURON model. This function can be created via the template provided by the GUI or loaded from a previously written file. The function must use the Python syntax of NEURON (with one exception: there can be no empty lines in the section which defines the names of the parameters) to access model elements, and allows the user to optimize a combination of model parameters or set parameters to a specific value, not known during the creation of the model (see use case 4). If such a function is defined, the GUI will hide the list of parameters, and direct selection of model parameters is no longer allowed. The function is checked against basic syntax errors, but we strongly recommend to double-check the function as other errors will be detected only during runtime.

### Loading a time-varying stimulus

The stimulation settings layer offers an option to load a time-varying stimulus, which will use the *play* method of NEURON to use the values in the given file to stimulate the model. The file should be a simple text file containing only the stimulation values. The number of values must be equal to the number of sample points generated by the simulation (one can calculate this by dividing the length of the simulation by the integration step size).
